# The Hansen's baccata #2 gene *Rvi12_Cd5* confers scab resistance to the susceptible apple cultivar “Gala Galaxy”

**DOI:** 10.1111/tpj.17214

**Published:** 2024-12-18

**Authors:** Ayesha Yousaf, Paolo Baldi, Stefano Piazza, Valeria Gualandri, Matteo Komjanc, Lorenza Dalla Costa, Andrea Patocchi, Mickael Malnoy

**Affiliations:** ^1^ Research and Innovation Centre, Fondazione Edmund Mach San Michele all'Adige Italy; ^2^ Department of Agricultural, Food, Environmental and Animal Sciences Università Degli Studi di Udine Udine Italy; ^3^ Technology Transfer Centre, Fondazione Edmund Mach San Michele all'Adige Italy; ^4^ Fruit Breeding Group, Department of Plant Breeding Agroscope Waedenswil Switzerland

**Keywords:** resistance genes, *Malus domestica*, gene cloning, apple pathogens, scab management, *Venturia inaequalis*

## Abstract

To enhance the breeding of new scab‐resistant apple cultivars, a comprehensive understanding of the mechanisms governing major scab resistance genes is essential. *Rvi12_Cd5* was previously identified as the best candidate gene for the *Rvi12* scab resistance of the crab apple “Hansen's baccata #2” by gene prediction and *in silico* analysis. In the present study, *Rvi12_Cd5* was used to transform the scab‐susceptible apple cultivar “Gala Galaxy.” Two constructs were prepared: the first carrying *Rvi12_Cd5* under the control of a 35S promoter and E9 terminator, and the second carrying *Rvi12_Cd5* under the control of its native promoter and terminator. All the transgenic lines were analyzed for T‐DNA integration, copy number, and expression of *Rvi12_Cd5* and phenotypically evaluated for scab resistance. The “Gala Galaxy” lines carrying the 35S promoter expressed *Rvi12_Cd5* at a high level, showing partial to high resistance against a mixed inoculum of *Venturia inaequalis*, with symptoms ranging from class 0 to 3b on the Chevalier scale. The transgenic lines carrying the native promoter showed a lower expression of *Rvi12_Cd5* compared with the 35S lines. Nevertheless, the low expression was sufficient to induce a resistance level comparable to that of the transgenic lines carrying the 35S promoter. These results indicate that *Rvi12_Cd5* confers scab resistance to a susceptible apple cultivar and that even a low level of gene transcript can trigger a plant response to *V. inaequalis* infection. After *HcrVf2* and *Vr2‐C*, *Rvi12_Cd5* is the third major apple scab resistance gene being functionally proven.

## Significance Statement

A new apple scab resistance gene was characterized. It may be a valuable resource for both breeders and scientists, to produce new apple cultivars that are naturally resistant to this dangerous pathogen.

## INTRODUCTION

Scab, caused by the fungus *Venturia inaequalis*, is one of the most important apple diseases worldwide (Gonzalez‐Dominguez et al., 2017). It mainly attacks fruits and leaves, causing serious damage in terms of fruit quality and marketability, with economic losses that can reach up to 70% of the entire production (Chane & Boyraz, 2017). In commercial orchards, apple scab is currently managed by 15–30 fungicides applications per year (Linhart et al., 2021). This can cause serious environmental problems and have a negative impact on human health (Kaiser et al., 2024). Moreover, plant pathogens may develop genetic resistance to fungicides, over time (Ayer et al., 2019). The use of cultivars carrying durable scab resistance can reduce the chemical control of the disease. In apples, more than 20 scab resistance loci are known (Khajuria et al., 2018), but to date, for only two of them the genes conferring apple scab resistance have been identified and functionally proven: *HcrVf2* (Belfanti et al., 2004) and *Vr2‐C* (Schouten et al., 2014), responsible for the *Rvi6* and *Rvi15* resistance, respectively.

The introgression of resistance genes into commercial cultivars can be achieved using two methods: traditional breeding and genetic transformation. Traditional breeding, using resistance genes from wild apples, is a long‐term process and is slowed down by the requirement of multiple generations of pseudo‐testcrossing and by the relatively long juvenile phase of apple trees (Peil et al., 2011). Therefore, it may take decades to obtain new resistant cultivars. Once resistance genes are available, genetic transformation is a much faster alternative. Nowadays, the modern plant breeding techniques, such as transgenesis and cisgenesis, allow the introgression of resistance genes, deriving from small‐fruited wild apple species, into susceptible commercial apple cultivars in a relatively short period of time (Szankowski et al., 2009; Vanblaere et al., 2014). Unfortunately, most of the scab‐resistant cultivars released on the market carry only *Rvi6*‐based resistance. This is problematic as, in recent years, strains of *V. inaequalis* capable of overcoming the *Rvi6*‐based resistance have emerged in Europe and the USA (Papp et al., 2020; Parisi et al., 2003). Therefore, it is of primary importance to identify and characterize new scab resistance genes. This would allow for generating new cultivars with more durable resistance by pyramiding multiple apple scab resistance genes in a single genotype using either classical breeding or genetic engineering.

The Siberian crab apple (*Malus baccata*) “Hansen's baccata #2” (HB2) is known to carry the *Rvi12* scab major resistance gene, which was mapped on linkage group 12 (LG12) (Erdin et al., 2006). The high level of scab resistance and very low number of resistance breakdowns was confirmed within the network of orchards in the frame of the VINQUEST project (Patocchi et al., 2020). In a previous work, *Rvi12* was fine mapped using a large segregating population derived from the cross “Gala” × “HB2” (Padmarasu et al., 2014). Starting from the markers bracketing the gene, a chromosome walk led to the identification of a single BAC clone spanning the *Rvi12* interval of the chromosome carrying the *Rvi12*. The BAC was sequenced and five putative candidate genes (*Rvi12_Cd1*, *Rvi12_Cd2*, *Rvi12_Cd3*, *Rvi12_Cd4*, *Rvi12_Cd5*) were identified (Padmarasu et al., 2018). All the candidates showed homology to known resistance genes. Nonetheless, *Rvi12_Cd1* and *Rvi12_Cd2* lacked two essential motifs (NB‐ARC and LRR) that are needed for pathogen recognition and activation of the downstream signaling pathway. As for *Rvi12_Cd3* and *Rvi12_Cd4*, they both contain the delay of germination 1 (DOG1) domain, that was previously shown to be involved in seed dormancy, but not in pathogen resistance (Bentsink et al., 2006). Only *Rvi12_Cd5* showing an N‐terminal signal peptide, an extracellular LRR repeats, a transmembrane domain, an intracellular domain containing nucleotide‐binding sites and a protein kinase domain with serine threonine specificity (Padmarasu et al., 2018), remained as first choice candidate for Rvi12 resistance, and consequently it was chosen for this study.

The aim of the present study was to verify whether *Rvi12_Cd5* confers apple scab resistance in the susceptible cultivar “Gala Galaxy.” To this end, we generated transgenic lines carrying the gene under the control of the cauliflower mosaic virus 35S constitutive promoter (35S‐P) or under the control of his native promoter. The obtained lines were then characterized for T‐DNA copy number (CN) and *Rvi12_CD5* mRNA expression and assessed for scab resistance after artificial inoculation with *V. inaequalis*.

## RESULTS

### Promoter analysis

The choice of the native promoter region had some limitations. In fact, *Rvi12_Cd5* was located on the outer margin of the BAC insert (Padmarasu et al., 2018), so only 906 bp of the genomic region upstream the coding sequence could be retrieved. Therefore, before the genetic transformation, the native promoter sequence of *Rvi12_Cd5* was analyzed, to find transcription factor‐binding motifs putatively involved in plant response to scab. The *Rvi12_Cd5* promoter sequence was compared with the first 900 bp of the promoters of the two other known apple scab resistance genes *HcrVf2* (Belfanti et al., 2004) and *Vr2‐C* (Schouten et al., 2014). Several motifs putatively involved in plant defense were found. The most represented motifs are given in Table 1. Only motifs showing at least one copy in each promoter were considered. The most represented motif was ARR1AT, with 11 copies in both *HcrVf2* and *Rvi12_Cd5*, while 5 copies were found in *Vr2‐C*. DOFCOREZM and CACTFTPPCA1 were also widely represented, the former with 5 copies in *HcrVf2* and *Rvi12_Cd5* and 6 copies in *Vr2‐C*, and the latter with 5 copies in *HcrVf2*, 3 copies in *Vr2‐C*, and 7 copies in *Rvi12_Cd5*. Multiple copies of GTGANTG10, CAATBOX1, WRKY71OS, and GATABOX were also found in all three promoters, while GT1CONSENSUS, WBOXNTERF3, MYBCORE, and IBOXCORE were still present in all the promoters but in some cases in a single copy.

**Table 1:** List of transcription factor‐binding motifs found in the promoter region of *HcrVf2* (Vf), *Vr2‐C* (Vr2), and *Rvi12_Cd5* (Vb)

Motif	Number of motifs				Function	Reference
	Vf	Vr2	Vb	Total		
ARR1AT	11	5	11	27	Cytokinin‐responsive	Chen et al. (2018)
DOFCOREZM	5	6	5	16	Hormone‐responsive	Benson et al. (2014)
CACTFTPPCA1	5	3	7	15	Mesophyll‐specific	Akyildiz et al. (2007)
GTGANTG10	5	3	4	12	Pollen‐specific	Zhao et al. (2022)
CAATBOX1	3	4	5	12	Tissue‐specific	Yang et al. (2021)
WRKY71OS	5	2	4	11	Disease resistance	Yang and Feng (2020)
GATABOX	2	2	7	11	Stress‐responsive	Zhu et al. (2019)
GT1CONSENSUS	3	4	1	8	Light‐responsive	Baek et al. (2016)
WBOXNTERF3	1	2	3	6	Wounding‐responsive	Nishiuchi et al. (2004)
MYBCORE	2	1	2	5	Abiotic stress‐related	Wang et al. (2019)
IBOXCORE	2	1	1	4	Light‐responsive	Meng et al. (2018)

### Genetic transformation

For the genetic transformation, a total of 1300 “Gala Galaxy” leaf explants were infected with *Agrobacterium tumefaciens* containing vector T (Figure S1) in two independent transformation rounds (transformations A, B; Table 2). In addition, 1350 “Gala Galaxy” leaf explants were infected with *A. tumefaciens* containing vector C (Figure S1) in two independent transformation rounds (transformations C, D; Table 2). From transformations A and B, 75 regenerants were obtained, while 60 regenerants were produced from transformations C and D approximately 6–7 months after *A. tumefaciens* transfection. Sixty‐two regenerants from transformations A and B and 60 regenerants from transformations C and D were tested by PCR to identify those with integrated T‐DNA and no *A. tumefaciens* contamination. Sixty lines from transformations A and B (T lines) and 45 lines from transformations C and D (C lines) showed integration of *Rvi12_Cd5* in their genome and no *A. tumefaciens* contamination (Table 2).

**Table 2:** Statistics of the *Agrobacterium tumefaciens*‐mediated transformations

Transformation	Vector	Infected leaves	Regenerants	PCR analysis	
				Tested	Positives^a^
A	T	450	15	4	4
B	T	850	60	58	56
C	C	450	15	15	15
D	C	900	45	45	30

^a^ Plants that showed the amplification of *Rvi2_Cd5* along the 35S promoter in vector T‐based transformants and solely *Rvi12_Cd5* in C‐based transformants after PCR screening were classified as a positive regenerants.

### Copy number and transcription level of Rvi12_Cd5

**Figure 1:**
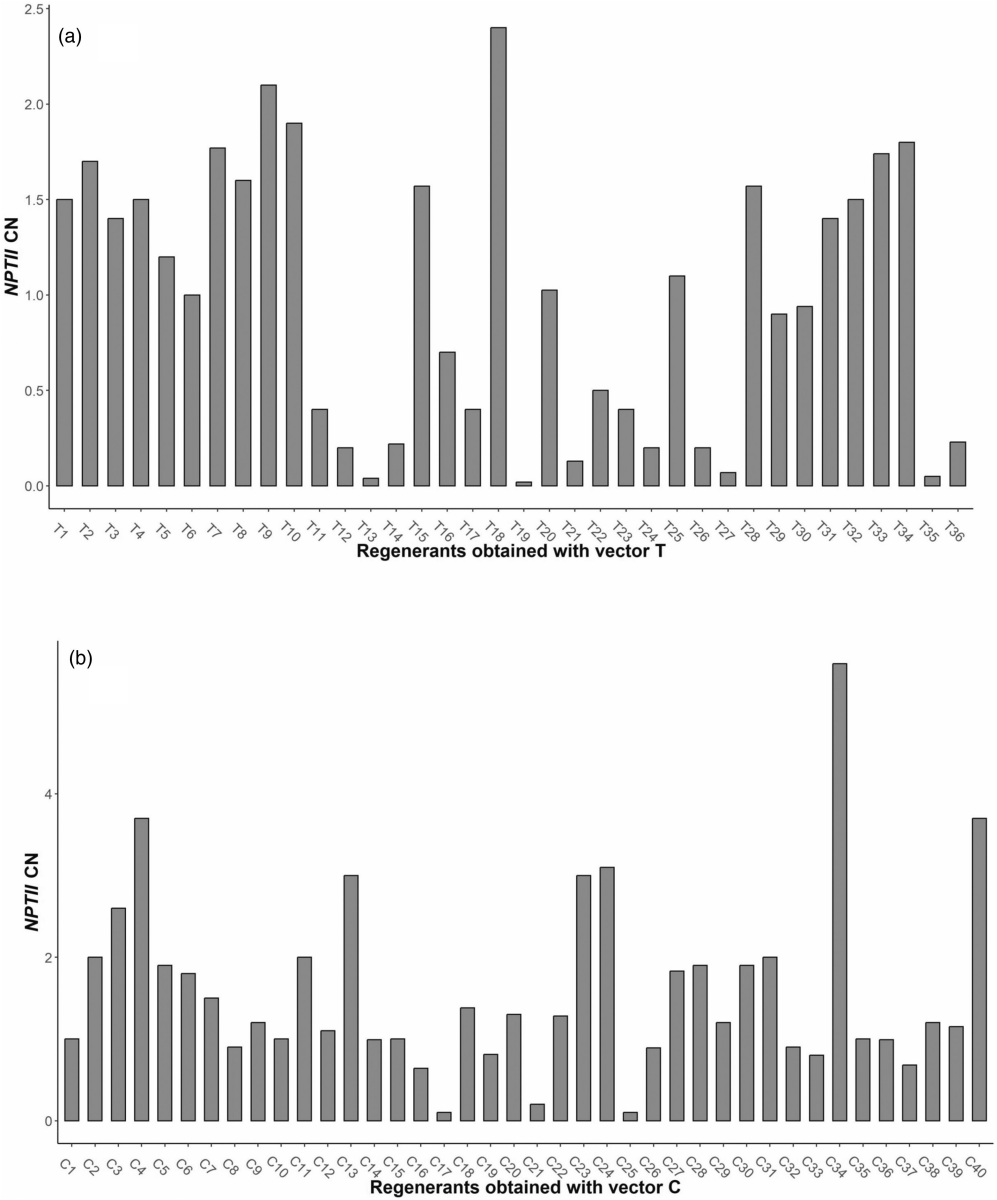
NptII copy number (CN) of transgenic lines. (a) Transgenic “Gala Galaxy” lines transformed with vector T (T lines). (b) Transgenic “Gala Galaxy” lines transformed with vector C (C lines). On the *y*‐axis, the copy number is indicated, as estimated by TaqMan real‐time PCR.

Quantitative real‐time PCR (qPCR) was used to randomly select and characterize a subset of 36 out of the 60 T lines and 40 out of 45 C lines available, for *nptII* integration CN. The *nptII* CN of the T lines ranged from 0.04 to 2.4 (Figure 1a), while the C lines showed CN ranging from 0.1 to 5.59 (Figure 1b). The 51 (29 T‐based transformants and 22 C‐based transformants) lines showing fractional CN were discarded as possible chimeras. Thus, the 20 lines (7 of T‐based transformants and 13 C‐based transformants) with *nptII* CN close to 1 and 2 were transferred from the shoot propagation medium to the rooting medium, and those that later produced roots were acclimatized to the greenhouse. After rooting, acclimatization, and CN confirmation, 5 T lines (T10, T20, T25, T29, and T30) and 12 C lines (C2, C5, C6, C8, C9, C10, C11, C12, C14, C15, C26, and C35) were selected for further analysis.

**Figure 2:**
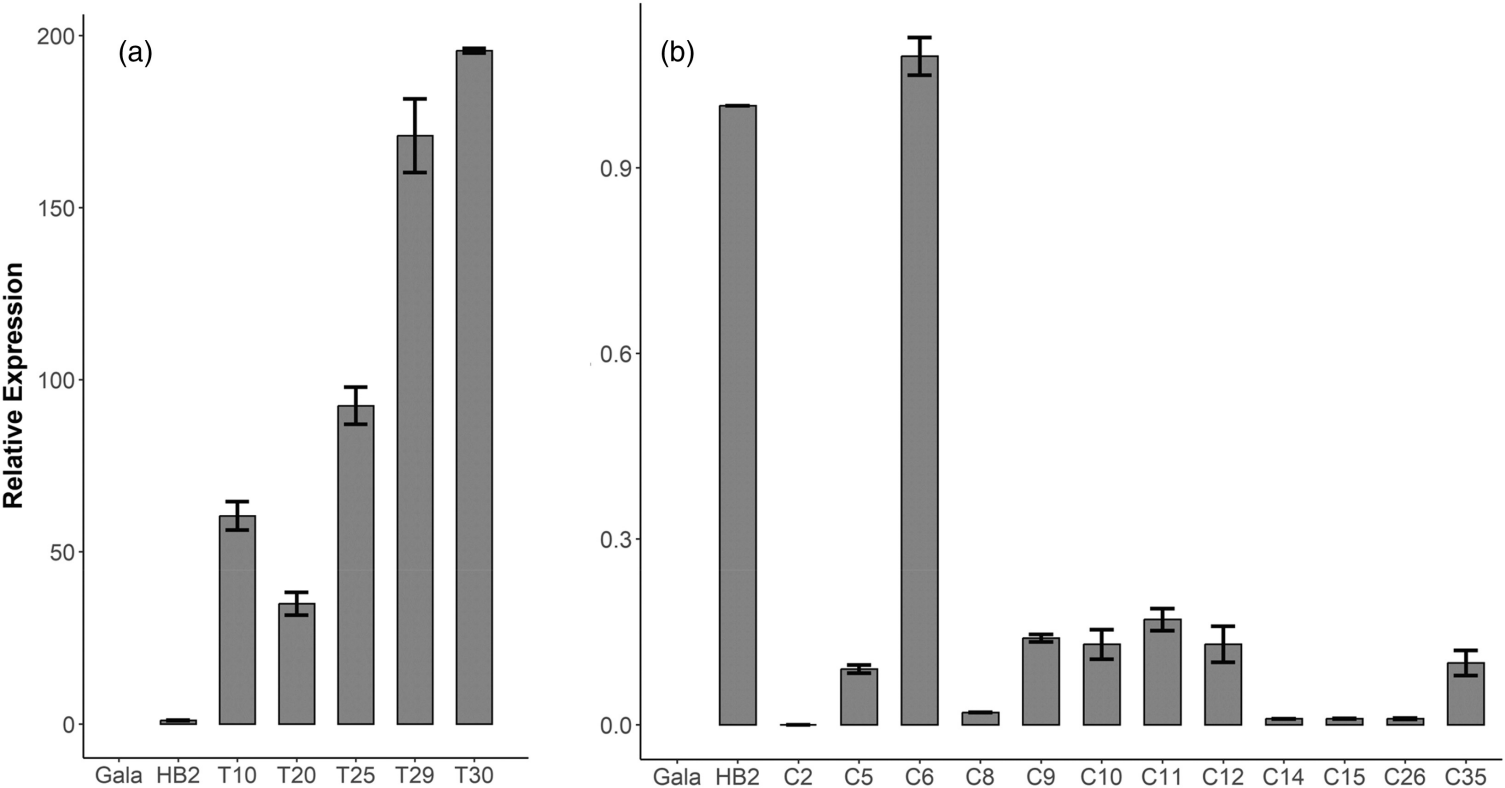
Expression levels of *Rvi12_Cd5* in “Gala Galaxy” (Gala), “HB2” and transgenic lines. (a) Transgenic “Gala Galaxy” lines transformed with vector T (T lines). (b) Transgenic “Gala Galaxy” lines transformed with vector C (C lines). The expression levels are indicated on the *y*‐axis and are relative to the value of “HB2.” All data were normalized to actin. The bars represent the standard deviations for three biological replicates.

The transcription level of *Rvi12_Cd5* was tested in all the selected lines. The T lines showed high *Rvi12_Cd5* transcription levels when compared with “HB2” (Figure 2a). Lines T29 and T30 showed transcription levels between 170 and 200‐fold higher than “HB2,” while lines T25 and T10 showed transcription levels 92‐fold and 60‐fold higher than “HB2,” respectively. Line T20 showed the lowest level of transcription, which was still 34‐fold higher than “HB2” (Figure 2a). On the contrary, the expression levels of most of the C lines were found to be 5‐ to 10‐fold lower than “HB2,” with some lines (e.g., C14, C15, and C26) showing barely detectable expression of *Rvi12_Cd5* (Figure 2b). The expression levels of lines C4 and C7 were twofold lower than “HB2,” while C6 was the only line that showed an expression level comparable to “HB2” (Figure 2b).

### Apple scab resistance evaluation

**Figure 3:**
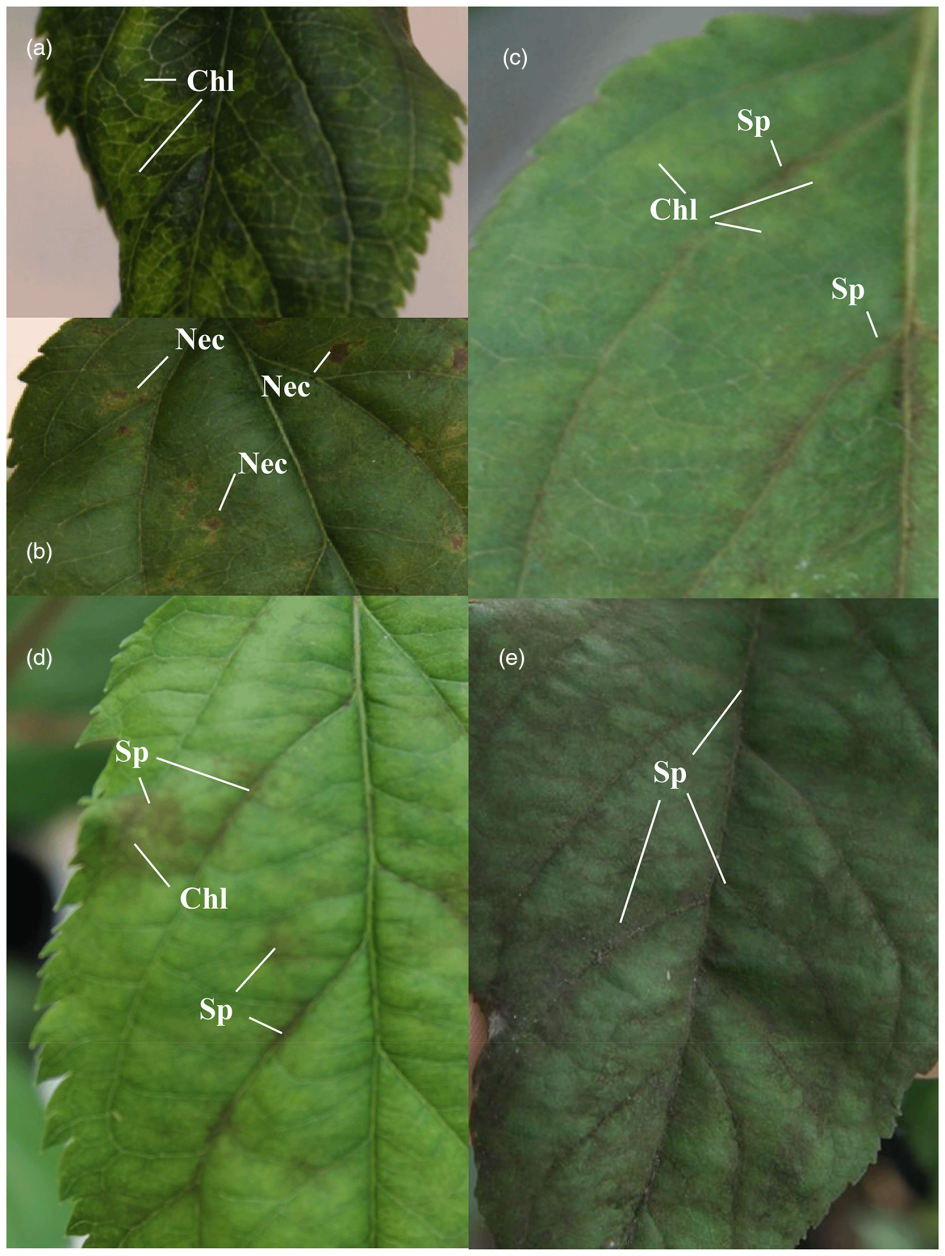
Classes of scab symptoms detected 21 days post‐inoculation (dpi). (a) Class 2, chlorosis (Chl). (b) Class 2, chlorosis with necrosis (Nec). (c) Class 3a, chlorosis (without necrosis in this specific case) with very light sporulation (Sp). (d) Class 3b, chlorosis (without necrosis in this specific case) with isolated sporulation. (e) Class 4, diffused sporulation. Leaves showing no visible symptoms were classified as class 0. Plants showing symptoms going from 0 to 3b were considered as resistant.

Five T lines (T10, T20, T25, T29, and T30) and 8 C lines (C5, C6, C8, C9, C10, C11, C12, and C35) with a relative expression level of *Rvi12_Cd5* higher than 0.01 were selected for scab resistance assessment. Symptoms were evaluated according to the Chevalier scale (Figure 3).

**Figure 4:**
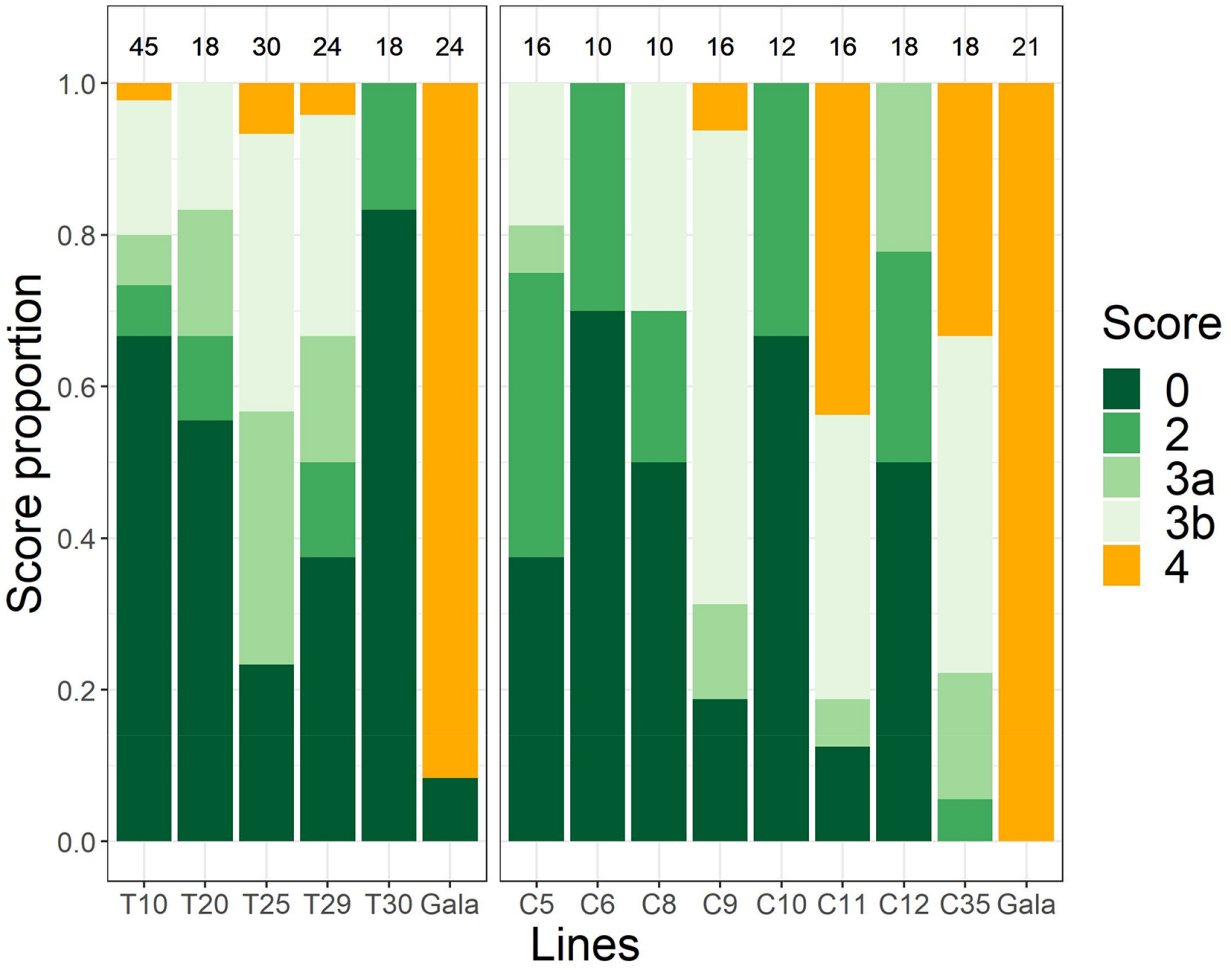
Graphical representation of the proportion of leaves of T and C lines (scored at 21 dpi) showing symptoms of resistance after inoculation with scab (green classes going from class 0 to 3b) or showing symptoms of susceptibility (orange, class 4). In the upper part of the figure, the number of evaluated leaves at 21 dpi is indicated for each line.

“Gala Galaxy,” the susceptible control, displayed abundant sporulation (class 4) covering most of the leaf in all experiments. Scab symptoms were already visible at 10–12 days post‐inoculation (dpi), intensifying up to 21 dpi. In no cases, any resistance reaction by the plant was observed on these leaves (Figure 4).

Two “Gala Galaxy” leaves showing no symptoms were detected, probably being escapes. For the transformed T lines, a range of symptoms was always detected among the different leaves evaluated, but generally, the overall resistance level of a single line was consistent among the different inoculations (Figure 4; Table 3).

**Table 3:** Results of the molecular characterization (copy number and transcription of the *Rvi12_Cd5* gene compared with “HB2”) and of the three apple scab inoculation trials of T and C lines at 21 dpi

Line or cultivar	CN	Transcription level compared with HB2	Infection trial 1						Trial 2						Trial 3					
			Plants	Leaves	Med	L	U	Max	Plants	Leaves	Med	L	U	Max	Plants	Leaves	Med	L	U	Max
T10	1.87	60.41	9	27	0	0	0	4	4	10	2	0	3b	3b	2	8	0	0	3a	3b
T20	0.95	34.92	2	6	0	0	0	3a	2	7	2	0	3b	3b	2	5	2	0	3a	3a
T25	1.12	92.44	5	15	3a	3a	3b	3b	2	6	3a	3a	3b	3b	3	9	3a	0	3b	4
T29	0.86	170.87	3	9	0	0	2	3b	3	9	3a	2	3b	4	2	6	3a	0	3b	3b
T30	0.94	195.63	2	6	0	0	0	0	2	6	0	0	2	2	2	6	0	0	0	0
Gala	NA	‐	4	12	4	4	4	4	2	6	4	4	4	4	2	6	4	4	4	4
C5	1.9	0.09	2	7	2	0	3b	3b	3	9	2	0	2	3a	‐	‐	‐	‐	‐	‐
C6	1.81	1.08	2	7	0	0	0	2	1	3	2	0	2	2	‐	‐	‐	‐	‐	‐
C8	0.85	0.02	1	3	3b	3b	3b	3b	2	7	0	0	2	2	‐	‐	‐	‐	‐	‐
C9	1.18	0.14	3	9	3b	3a	3b	4	2	7	3b	0	3b	3b	‐	‐	‐	‐	‐	‐
C10	1	0.13	3	9	0	0	2	2	1	3	2	0	2	2	‐	‐	‐	‐	‐	‐
C11	1.98	0.17	3	9	4	3b	4	4	2	7	3b	0	3b	3b	‐	‐	‐	‐	‐	‐
C12	1.06	0.13	3	9	2	0	3a	3a	3	9	0	0	2	3a	‐	‐	‐	‐	‐	‐
C35	0.97	0.1	3	9	4	3b	4	4	3	9	3b	3a	3b	3b	‐	‐	‐	‐	‐	‐
Gala	NA	‐	4	12	4	4	4	4	3	9	4	4	4	4	‐	‐	‐	‐	‐	‐

The median values of symptoms were determined using data from all observed leaves (3–4 leaves per plant) from all plants within each line at each time point of the infection trail. Symptom assessment according to the scab classes described by Chevalier et al. (1991).

L, lower quartile (Q1); Max, maximum value observed; Med, median (Q2); U, upper quartile (Q3).

In all three inoculation trials, the median of all the lines was always lower than class 4, the only class considered as a sign of susceptibility. Line T30 demonstrated high resistance to *V. inaequalis*. In most cases, it did not show any visible symptoms (class 0). On some leaves, this line exhibited chlorotic lesions, sometimes with very small necrosis in the center (class 2; Figure 4; Table 3). The T‐line showing the most severe scab symptoms, but still not susceptible, was line T25, which in all three experiments had a median of class 3a (Table 3). In a few cases, lines T10, T25, and T29 showed class 4 symptoms (Table 3; Table S1).

In the C lines a range of symptoms was detected, with an overall good consistency in the resistance level of each line between the two repetitions (Figure 4; Table 3). The susceptible control “Gala Galaxy” always showed complete susceptibility, with distinct abundant sporulation (class 4). By contrast, lines C6 and C10 appeared to be highly resistant (median in both experiments of max class 2), as all the leaves were class 0 and class 2. Line C12 showed a high resistance level, with most of the leaves classified as class 0 or class 2 and only a few leaves showing class 3a (Figure 4; Table 3). Lines C5 and C8 showed an intermediate level of resistance, with symptoms ranging from 0 to 3b, while lines C9 showed only partial resistance, with most of the leaves showing class 3b and, in one case, even class 4 (Figure 4; Table S1). C11 and C35 were the two lines showing the lowest levels of resistance. Both lines had class 4 and class 3b as the median in the two experiments (Figure 4; Table 3).

**Figure 5:**
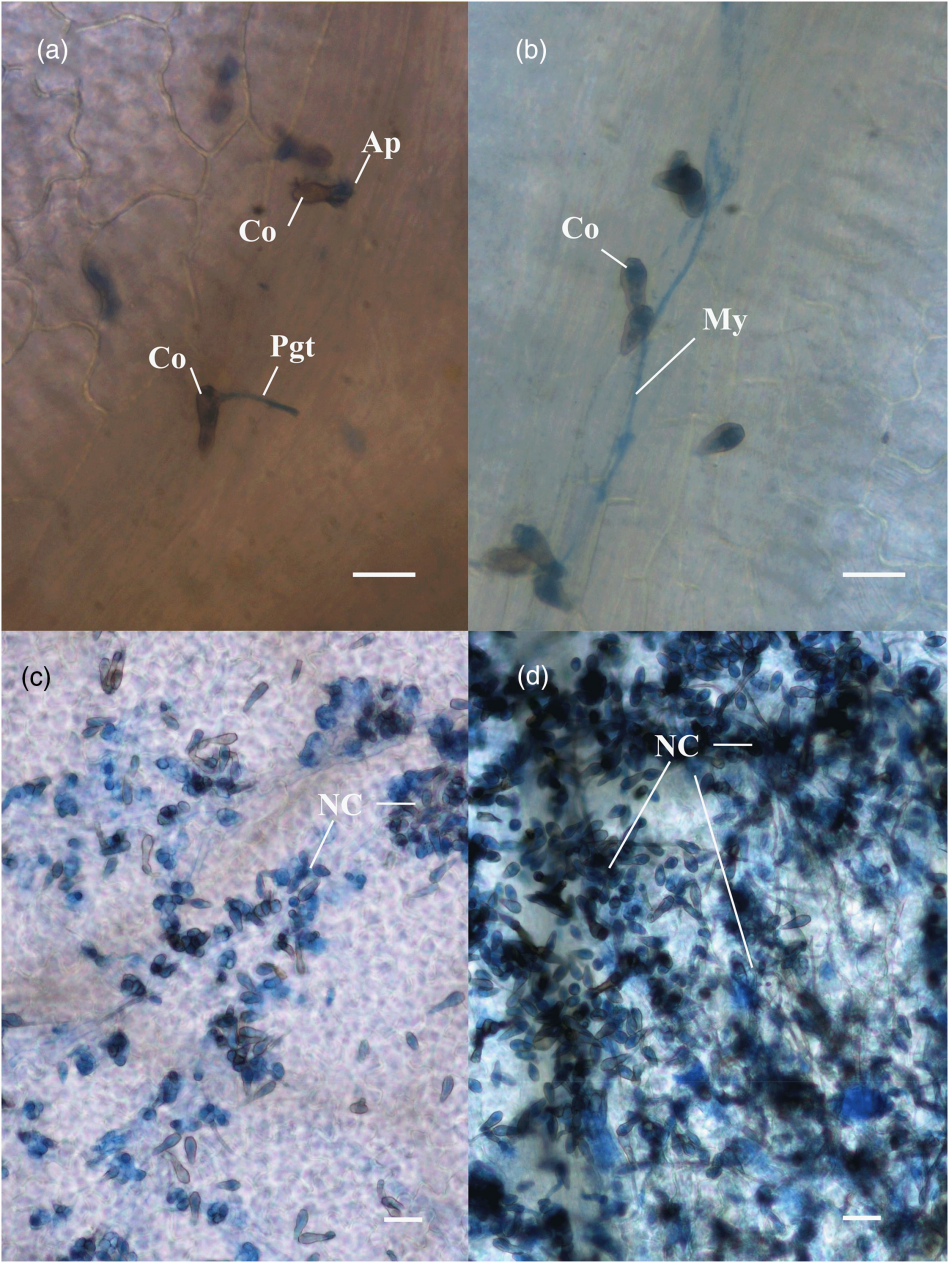
Microscopic characterization of *V. inaequalis* conidia on “Gala Galaxy” and transgenic plants showing different levels of resistance. (a) Germinated conidia (Co) with appressorium (Ap) and primary germtube (Pgt), typical of class 0 leaves. (b) Germinated conidia producing a short mycelium (My), typical of class 2 leaves. (c) Germinated conidia producing mycelium and sparse sporulation with low production of new conidia (NC), typical of class 3a and 3b leaves (the difference was only in the sporulation intensity, lower in 3a than in 3b). (d) Germinated conidia producing mycelium and abundant sporulation with high production of new conidia, typical of class 4 leaves (“Gala Galaxy”). Only class 4 was considered to be associated to susceptibly as only in this class no (resistance) reaction of the plant was observed. The scale bar is 20 μm.

All the leaves were evaluated at the microscopic level to confirm the presence of germinated *V. inaequalis* conidia. On the leaves classified as class 0, numerous germinated conidia were found, but all were blocked at a very early stage of development, showing only the development of an appressorium and a short primary germination tube (Figure 5a). On the apple leaves classified as class 2, some of the germinated conidia were capable of producing a short mycelium, but the growth was always very weak, and in no case were sporulating conidiophores observed (Figure 5b). The microscopic analysis of leaves classified as class 3a and 3b showed very similar results. Numerous conidia were capable of germinating and producing mycelial stroma. Nonetheless, the growth of the fungus was usually quite weak, resulting in sparse or scattered sporulating stroma with limited production of new conidia (Figure 5c). Finally, on the leaves classified as class 4, vigorous, and quite homogeneous growth of mycelium was observed, with abundant sporulation and production of new conidia (Figure 5d).

## DISCUSSION

### Rvi12_Cd5 confers scab resistance

The presented data indicate that *Rvi12_Cd5* is sufficient to confer apple scab resistance to the susceptible apple cultivar “Gala Galaxy,” as all the transformed lines analyzed showed higher resistance levels than the untransformed plants. The macroscopic and microscopic evaluations of the phenotypes were consistent. Microscopic analysis showed germinated conidia, therefore excluding the possibility that the resistance of the transformed lines was due to the low vitality of the inoculum or inoculum escapes. The degree of resistance in the T and C lines was always variable. Most of the transformed lines ranged from full resistance with almost no visible symptoms (lines T30, C5, C6, C10, and C12) to weaker resistance with limited sporulation (class 3a and 3b; lines T10, T20, T25, T29, C8, and C9). In six cases, few leaves of some transgenic lines showing class 4 symptoms were detected (lines T10, T25, T29, C9, C11, and C35).

Such variability of resistance in transgenic lines with the same genetic background is difficult to explain. Many factors may be involved. From the literature, it is known that apple plants carrying *Rvi12* may display a wide range of resistance classes, going from 0 to 3b, with only class 1 (pinpoint) missing (Chevalier et al., 1991; Erdin et al., 2006; Padmarasu et al., 2014). In a previous study, most of the resistant plants (54 out of 76; 71%) belonging to a segregating population derived from the cross “Golden Delicious” × “HB2” were classified as class 3b, indicating that the level of resistance conferred by *Rvi12* in some cases may be quite low. Moreover, the integration site of T‐DNA during genetic transformation is random. Therefore, a possible factor contributing to the variation in resistance among different transgenic lines is the so‐called positional effect, that is caused by variables such as chromatin‐based regulation, methylation level, and different regulatory elements that may be present around the insertion point (Abdulraheem et al., 2024). Post‐transcriptional mechanisms may also be involved (Gong et al., 2022).

According to our data, the resistance level of each transgenic line does not depend directly on the constitutive transcription level of *Rvi12_Cd5*, as line C6 showed the same phenotype as line T30, with an expression level that was about 200 times lower. By contrast, line T29 presented a *Rvi12_Cd5* expression level very similar to line T30, but showed a lower level of resistance, with a predominance of class 3a and 3b symptoms. Moreover, lines C10 and C12 showed a high level of resistance with a *Rvi12_Cd5* expression level that was approximately five times lower than that of line C6 and almost 1000 times lower than in line T30. Taken together, these findings seem to indicate that even a low *Rvi12_Cd5* gene expression level is sufficient to trigger the defense response of the plant. This is in accordance with previous results showing significant scab resistance due to the low expression level of *HcrVf2* (Vanblaere et al., 2014). It is worth noting that we assessed only the constitutive expression level of *Rvi12_Cd5* in the uninoculated plants in this study. Therefore, it is possible that the level of expression of *Rvi12_Cd5* in both C and T lines may increase following *V. inaequalis* infection, as previously found for *HcrVf2* (Malnoy et al., 2008).

*Rvi12_Cd5* is a typical pattern recognition receptor, responsible for the so‐called pattern‐triggered immunity (PTI) of plants (Saijo et al., 2018). Similarly to the rice resistance gene *Xa21* (Song et al., 1995), *Rvi12_Cd5* showed all the domains that are essential to induce PTI. In particular, *Rvi12_Cd5* presents an extracellular leucine‐rich repeat domain for pathogen recognition, a transmembrane domain and a cytoplasmic kinase domain. After pathogen recognition, the kinase activation triggers several signaling pathways, such as the mitogen‐activated protein kinases (MAPK) kinase cascade, an influx of Ca^2+^ into the cytosol, and production of reactive oxygen species (Saijo et al., 2018).

Even though *Rvi12_Cd5* appeared to be active in conferring scab resistance, four additional candidates resistance genes were identified in the same genomic region (Padmarasu et al., 2018). Sequence analysis revealed that all those genes seemed to lack some essential domains to be active as scab resistance genes, but no functional analysis was performed (Padmarasu et al., 2018). Therefore, the possible role of one or more of these genes in apple scab resistance cannot be fully excluded yet.

### Gene regulation

The 900 bp native promoter used in our study seemed to be enough to ensure the expression of *Rvi12_Cd5*, even though some of the C lines showed a very low level of expression. The correlation between promoter length and the amount of transcribed RNA is not always linear, and it depends on the type and number of regulatory elements present on the promoter itself. In previous studies on the *HcrVf2* promoter, contradictory results were sometimes found. Joshi et al. (2011) used a long promoter (2000 bp) and a shorter one (288 bp) in transformation experiments with *HcrVf2* on the susceptible cultivar “Gala.” The performances of the two promoters were not found to be different. On the contrary, Szankowski et al. (2009) found differences in *HcrVf2* expression depending on the length of the promoters used (115, 288, or 779 bp). In particular, the transgenic lines carrying the 115 bp promoter, showed only a weak resistance, while those carrying the 288 and 779 bp promoters showed very similar gene expression level as well as scab resistance, indicating that a native promoter of 288 bp is already sufficient to confer complete resistance.

The comparison of the promoter regions of *Rvi12_Cd5*, *HcrVf2*, and *Vr2‐C* shows several common regulatory elements that could be related to plant defense. The most represented motif (ARR1AT) is a cytokinin‐responsive element (Chen et al., 2018). Cytokinins are plant hormones that are known to play a role in plant immunity by an elevation of salicylic acid‐dependent defense response (Watson & Argueso, 2017). The second most abundant motif (DOFCOREZM) is a hormone‐responsive element that was found to be very common among disease resistance genes across six plant species (Benson et al., 2014). Another common disease resistance motif found in all three genes, *Rvi12_Cd5*, *HcrVf2*, and *Vr2‐C*, was WRKY71OS, which plays an important role in early defense response mediating fungal elicitor‐induced gene expression (Eulgem et al., 1999; Yang & Feng, 2020). Two other motifs (GATABOX and MYBCORE) are known stress‐responsive elements (Wang et al., 2019; Zhu et al., 2019), while GT1CONSENSUS and IBOXCORE are thought to be related to light response in plants (Baek et al., 2016; Meng et al., 2018). Further, the length of the terminator sequence could be involved in modifying the gene expression level (Dean et al., 1989). Compared with the terminator length (1506 bp) used in the present study, shorter terminator lengths (1 kbp, 437–480 bp, and 220 bp) were used by Malnoy et al. (2008), Joshi et al. (2011), and Vanblaere et al. (2014), which contributed to low, medium, and very low *HcrVf2* mRNA expression, respectively. Whether the lengths of both the natural promoter and terminator influence the expression of *Rvi12_Cd5* needs to be clarified in future studies.

## CONCLUSIONS

Most apple cultivars on the market are highly susceptible to *V. inaequalis*. The frequent application of chemicals that modern apple growth requires is problematic from both economic and environmental points of view. The functional characterization of the third scab resistance gene provides several opportunities for the improvement of more sustainable apple production. First, it allows the development of molecular markers that are perfectly linked to resistance and that could be used in marker‐assisted selection. Second, *Rvi12_Cd5* could be introgressed with *HcrVf2* and *Vr2‐C* in a commercial apple cultivar, for example, by a cisgenic approach (Vanblaere et al., 2014). This is not possible to achieve by conventional breeding, due to apple self‐incompatibility that does not allow backcrossing (Wu et al., 2013). The existence of apple transformed lines carrying different scab resistance genes in the same genetic background of “Gala” (Belfanti et al., 2004; Schouten et al., 2014) additionally provides a unique research opportunity for understanding the molecular basis of scab resistance and studying the pathways activated by the different resistance genes. *Rvi12_Cd5* is the third apple scab resistance gene identified thus far, therefore representing a valuable resource from both a scientific and commercial point of view.

## MATERIALS AND METHODS

### Construct preparation and genetic transformation

The two binary vectors used in this work were designed and assembled by a DNA cloning service (Hamburg, Germany). In vector D301p9oU10‐35S‐PK123 (Vector T), the coding sequence of *Rvi12_Cd5* (3352 bp) from “HB2” was under the control of the cauliflower mosaic virus 35S promoter (35S‐P) and E9 terminator (Figure S1a). In vector D308pBNpt‐MdPromotvb (vector C), the native control sequences of *Rvi12_Cd5* were used, that is, the first 903 bp of the native promoter and 1506 bp of the native terminator (Figure S1b). The vectors were transferred into *A. tumefaciens* strain EHA105 competent cells carrying the helper plasmid pCH32, by electroporation (Chetty et al., 2013; Hood et al., 1993). *A. tumefaciens‐*mediated transformation of apple was performed using the protocol described by Vanblaere et al. (2011) with minor modifications: myo‐inositol and 6‐benzylaminopurine were utilized exclusively in the coculture and regeneration media; the shoot propagation medium was prepared according to Joshi et al. (2009), while the rooting medium was prepared according to Szankowski et al. (2003).

Explants were prepared using the top four youngest leaves of *in vitro* propagated shoots of “Gala Galaxy”. After 6–7 months of coculture with *Agrobacterium*, regenerants were multiplied *in vitro* by transferring to the shoot propagation medium. Regenerating shoots were tested for T‐DNA insertion. Approximately, 100 mg of leaves were used for DNA extraction (Nucleospin® Plant II, Mini kit; Macherey–Nagel, Düren, Germany). After quantification by NanoDrop 8000 Spectro‐photometer (Thermo Scientific, Inc., Bedford, MA, USA), the samples were diluted to a final concentration of 50 ng μl^−1^. Polymerase chain reactions (PCRs) were performed to confirm the presence of *Rvi12_Cd5* along the 35S promoter in the case of vector T‐based transformants and solely *Rvi12_Cd5* in vector C‐based transformants. PCRs were performed using a thermocycler‐3000 (Biometra), GoTaq Green Master Mix 2X (Promega, Fitchburg, MA, USA), 1.5 μl of genomic DNA, and 10 μm of each primer (Table S2). The PCR cycling conditions for the 35S promoter were: 2 min at 95°C, followed by 35 cycles at 95°C for 30 sec, 58°C for 30 sec, and 72°C for 30 sec, with a final extension of 5 min at 72°C. The PCR cycling conditions used for *Rvi12_Cd5* were those of the 35S promoter, with the exception of the annealing temperature, which was 61°C for 30 sec. Confirmation of the absence of *A. tumefaciens* in the transformants was also performed by PCR, according to Herzog et al. (2012), using primers for the virulence gene (Table S2), with cycling conditions as used for the 35S promoter.

### Promoter analysis

The regions upstream of the start codon of *Rvi12_Cd5* with a range of −900 bp were considered putative regulatory regions. The corresponding regions of *HcrVf2* (Belfanti et al., 2004) and *Vr2‐C* (Schouten et al., 2014) were retrieved. Selected promoter regions were then subjected to the NewPLACE transcription factor database, retrieving the transcription factor‐binding information (Higo et al., 1999).

### Copy number assessment

The insertion CN was estimated by quantifying the number of *nptII* segments present in positive transformants by TaqMan real‐time PCR according to Dalla Costa et al. (2019). The primers used are shown in Table S2.

### Rooting and acclimatization of transgenic plants to greenhouse

*In vitro* shoots of transgenic “Gala Galaxy” lines showing *nptII* CN close to 1 and 2 were transferred from the shoot propagation medium to the rooting medium. Rooted plants were transferred to a plastic tray with individual soil‐filled compartments (“Terriccio Vegetal Radic” – TerComposti S.p.a., Brescia, Italy), covered by a plastic sheet on the top to preserve humidity and kept in a growth chamber at 24 ± 1°C, 16/8‐h light/dark period, relative humidity of 70% ± 5%. After 3 weeks, the plastic covering was progressively opened to allow for a gradual decrease in humidity (Pessina et al., 2016). After another 1–2 weeks, the plants were repotted into 0.25 L plastic pots and moved to the greenhouse under the same conditions used for the growth chambers.

### Expression analysis

RNA extraction from about 100 mg of frozen leaf tissue was performed using the Spectrum plant total RNA kit (Sigma‐Aldrich, St. Louis, MO, USA), following the manufacturer's instructions. The Invitrogen SuperScript cDNA Synthesis Kit (Thermo Fisher Scientific, Inc.) was used for cDNA synthesis. RNA and cDNA quantification were obtained by a NanoDrop 8000 spectrophotometer (Thermo Fisher Scientific, Inc.). All the cDNA samples were diluted to a final concentration of 50 ng μl^−1^. Specific primers for *Rvi12_cd5* and actin were designed (Table S2) using the online software Primer3Plus (Untergasser et al., 2007). The qPCR was performed using Fast SYBR green master mix (Life Technologies, Carlsbad, CA, USA). The reactions were loaded on a ViiA7™ instrument (Life Technologies). The qPCR reactions were carried out using the following thermal conditions: initial incubation at 95°C for 20 sec; 40 cycles at 95°C for 15 sec and 60°C for 30 sec. All experiments were the result of three independent biological replicates. Relative gene expression values were obtained using the Delta–Delta CT method (Livak & Schmittgen, 2001). Data were normalized using actin as a housekeeping gene (Perini et al., 2014).

### Scab inoculation

Acclimatized transgenic and wild‐type ‘Gala Galaxy’ lines were used for inoculation experiments. For T lines (developed using vector T), 6–15 plants per line were used in three independent inoculation experiments. For C lines (developed using vector C), 3–8 plants per line were used in two independent inoculation experiments. Three to four top young leaves of each actively growing plant were inoculated by spraying a mixed suspension of *V. inaequalis*, obtained from a natural population collected in the field (5 × 10^5^ conidia ml^−1^ with a germination rate of 80–90%). Conidia concentration was measured by a hemocytometer (Fisher Scientific, Hampton, NH, USA). Plants were kept in controlled conditions for 48 h (16‐h photoperiod of 40 μmol m^−2^ sec^−1^, 18 ± 1°C, and 100% relative humidity [RH]), then growth for 10 days at 60% RH and again at 100% RH for 2 days. The assessment of the symptoms was performed after 21 days of growth at 60% RH.

### Symptom evaluation

Macroscopic symptoms were evaluated on 3–4 inoculated leaves per plant 21 days post‐inoculation (dpi), according to the Chevalier scale (Chevalier et al., 1991), with some modifications. Leaves free of symptoms were classified as class 0; leaves showing chlorotic spots, sometimes with a small necrosis but no sporulation, were classified as class 2 (Figure 3a,b); leaves showing chlorosis and or necrosis with very light sporulation (usually along the leaf veins) were classified as class 3a (Figure 3c); leaves showing chlorosis and or necrosis with isolated sporulation were classified as class 3b (Figure 3d); and leaves showing diffused sporulation on all the surface and no signs of chlorosis/necrosis were classified as class 4 (Figure 3e). The macroscopic symptoms and their median and confidence limits were analyzed to describe the overall response of each transgenic line and control (Belfanti et al., 2004). Optical microscopy analyses were performed 22 dpi. The leaf samples for microscopy were prepared using the protocol described by Bénaouf and Parisi (1998). Briefly, 10–27 leaf discs per line and control were harvested and incubated at 75°C for 30 min in a solution of 96% ethanol and 4% acetic acid to decolorate them. Leaf discs were then incubated for 7 min in a staining solution (70% ethanol, 29.9% water and 0.1% Aniline‐blue lactophenol) at 75°C. After three washes with 70% ethanol, leaf discs were incubated for 2 h at room temperature in a conservation solution (50% ethanol and 50% lactic acid).

## AUTHOR CONTRIBUTIONS

AY: Investigation, Data curation, Writing – review and editing; PB: Conceptualization, Methodology, Writing – original draft, Writing – review and editing; SP: Investigation; VG: Investigation; MK: Investigation; LDC: Investigation, Writing – review and editing; AP: Conceptualization, Methodology, Writing – review and editing; MM: Conceptualization, Methodology, Writing – review and editing.

## CONFLICT OF INTEREST

The authors declare no conflicts of interest.

## Supporting information


**Figure S1.** Map of the 14 kb D301P9OU10‐35S‐PK123 binary vector (vector T) containing the gene *Rvi12_Cd5* (ORF1) controlled by 35S promoter used for apple ‘Gala Galaxy’ transformation.

**Table S1.** Scab infection raw data showing symptom evaluation of all the leaves tested.

**Table S2.** List of all primers and probes used.

## Data Availability

The sequence of *Rvi12_Cd5* was submitted to GenBank with the following accession number: BankIt2837882 Rvi12_cd5 PP897949.
