# Supplementary material for: The Hansen's baccata #2 gene Rvi12_Cd5 confers scab resistance to the susceptible apple cultivar “Gala Galaxy”
Source: Plant J. 2024 Dec 18;121(2):e17214. doi: 10.1111/tpj.17214 (PMC11776037; doi:10.1111/tpj.17214)

**Fig. S1.** **A** Map of the 14 kb D301P9OU10-35S-PK123 binary vector (**vector T**) containing the gene *Rvi12_Cd5* (ORF1) controlled by 35S promoter used for apple ‘Gala Galaxy’ transformation. Colicin E1 and pVS1 origins of replication (ColE1 & pVS1); streptomycin/spectinomycin resistance genes (Sm/Sp); left and right borders (LB & RB); Cauliflower Mosaic Virus 35S promoter (35S-P); Neomycin phosphotransferase II (nptII); E9 terminator (E9); Arabidopsis thaliana Ubiquitin-10 promoter (Pro-Ubq10At); terminator element as octopine synthase gene (OCS). **B** Map of the 16 kb binary vector D308pBNpt-MdPK1-3 (**vector C**) containing the gene *Rvi12 Cd5* (ORF1) controlled by its native promoter (NP) and native terminator (NP) used for apple ‘Gala Galaxy’ transformation. Native promoter, open reading frames of apple scab resistance gene *Rvi12_Cd5* and native terminator (NP, ORF-(1-3) & NT) from the Siberian crab apple ‘Hansen’s baccata #2’(HB2); origins of replication and basis of mobility (Colicin E1 & BOM); streptomycin/spectinomycin resistance genes (Sm/Sp); left and right borders (LB & RB); Cauliflower Mosaic Virus 35S promoter and terminator (35S-P & 35S-T); Neomycin phosphotransferase II functions as kanamycin resistance gene for positive transformation selection (*nptII*); terminator element as octopine synthase gene (OCS); site-specific recombinase in vector pB (Resolvase); the partition ATPase (ParA); the centromere-binding protein (ParB); Enhancer element (Stsl1); Heat shock promoter of the soya bean gene (HSP); flippase gene (FLP); Flippase Recognition Target sites (FRT).

**A**


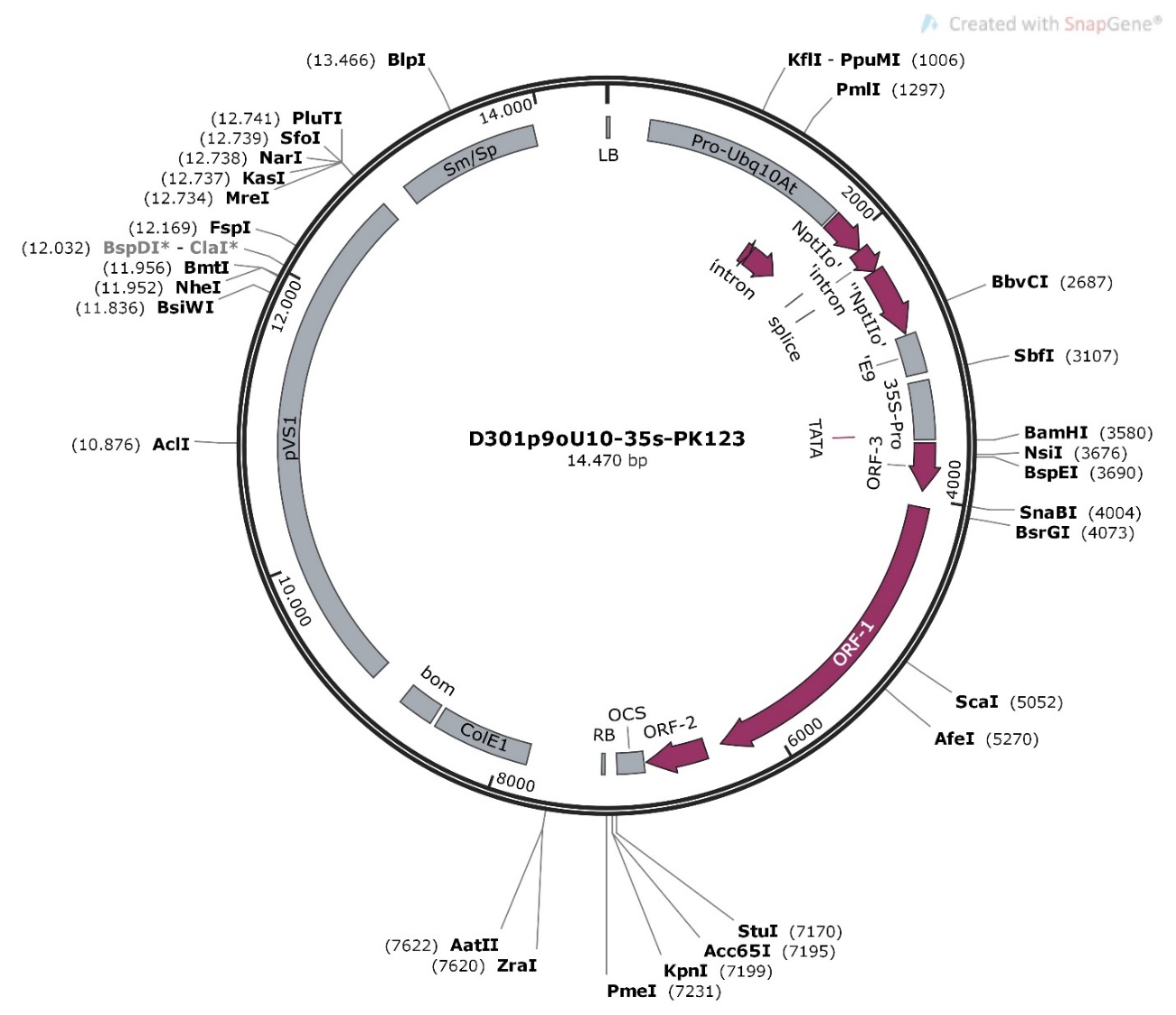


**B**


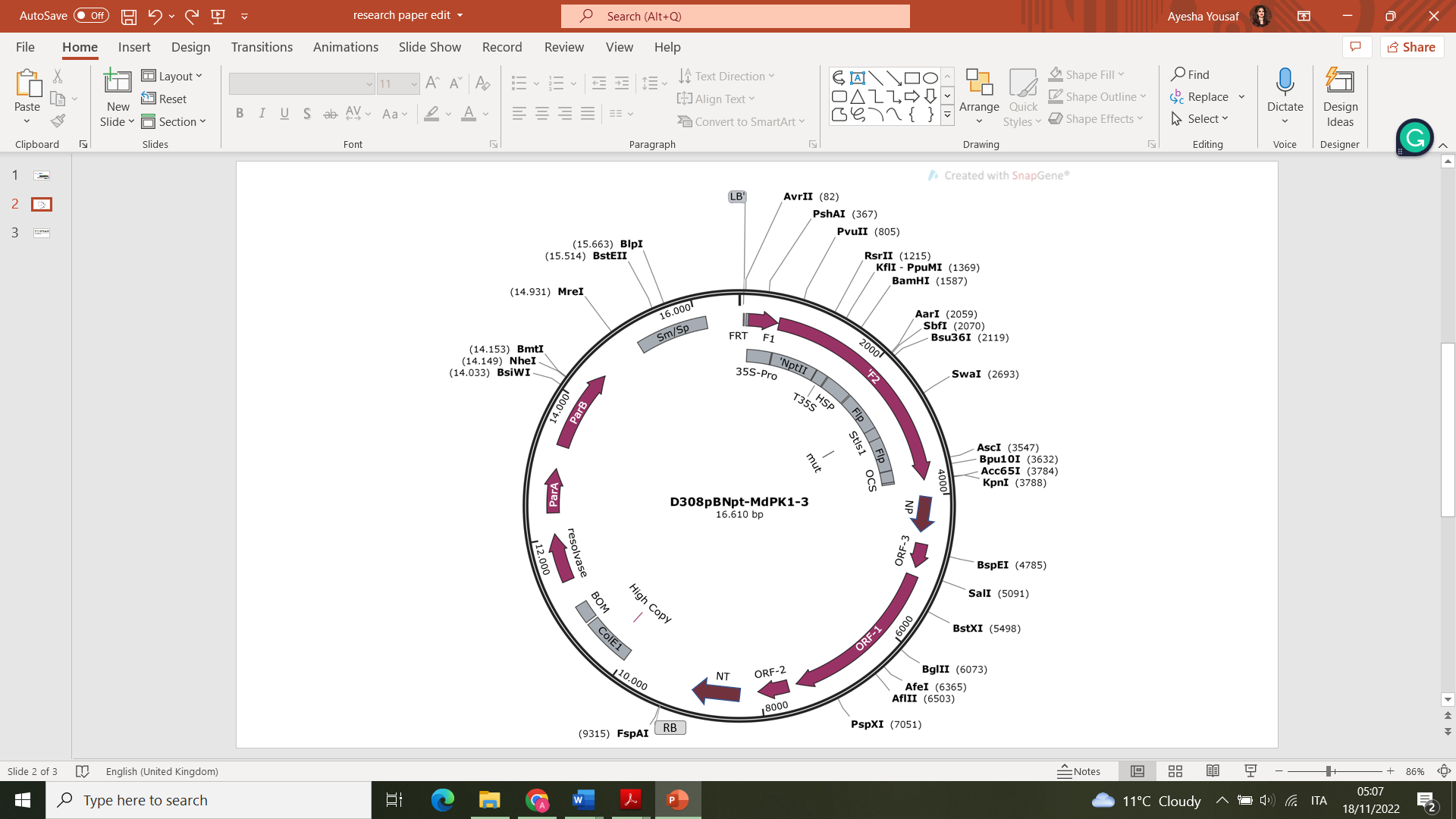

Supplement: Supplementary file 1 — Figure S1. Map of the 14 kb D301P9OU10‐35S‐PK123 binary vector (vector T) containing the gene Rvi12_Cd5 (ORF1) controlled by 35S promoter used for apple ‘Gala Galaxy’ transformation. [file TPJ-121-0-s003.docx]
