# Supplementary material for: The Hansen's baccata #2 gene Rvi12_Cd5 confers scab resistance to the susceptible apple cultivar “Gala Galaxy”
Source: Plant J. 2024 Dec 18;121(2):e17214. doi: 10.1111/tpj.17214 (PMC11776037; doi:10.1111/tpj.17214)
Supplement: Supplementary file 3 — Table S2. List of all primers and probes used. [file TPJ-121-0-s002.docx]

**Table S2.** List of all primers and probes used.

| **Primer and probe name** | **Sequence (5’-3’)** | **Amplicon length (bps)** | **Annealing temperature (°C)** | |
| --- | --- | --- | --- | --- |
| **Identification of transformants carrying vector T**  C_26_35S  PK123_R1 | F: 5′GCTATCGTTCAAGATGCCTCT3′  R: 5′CAATGGAAGGGAATGGAGCC3′† | 1572 | | 58 |
| **Identification of transformants carrying vector C**  SY170612171-093_F  SY170612171-093_R  ***NptII* copy number**  MdTOPO6 | F: 5′GGAGTCTTAAACTTGCCCGC3′†  R: 5′CAGAACCAAAACTTCCCGTG3′†  F: 5′TGTGGAAGGAGATCAAAGCGCA3′§  R: 5′CGCGTTGCTTCTTTGCTGCA3′§ | 1574  196 | | 61  58 |
| MdTOPO6_probe | FAM-5′-ACATGCCAACAGGAACAATCACA-3′-TAMRA§ |  | |  |
| NPTII | F: 5′CTTGCCGAATATCATGGTGGAA3′§  R: 5′GGTAGCCAACGCTATGTCCTGA3′§ | 100 | | 58 |
| NPTII_probe  ***Rvi12_Cd5* expression analysis**  MdACT_F  MdACT_R  Rvi12_Cd5_F2  Rvi12_Cd5_R2  **Colony PCR**  Rvi12_Cd5_F  Rvi12_Cd5_R  **Virulence gene for *Agrobacterium* contamination** | FAM-5′-TTCTGGATTCATCGACTGTGGC-3′-TAMRA§  F: 5′TGACCGAATGAGCAAGGAAATTACT3′$  R: 5′TACTCAGCTTTGGCAATCCACATC3′$  F: 5′GAGTCTTAAACTTGCCCGC3′†  R: 5′CCTGTGAAATTATTCATACAAC3′†  F: 5′ATGATGGAGCATTCACGTACTATTCG3′†  R: 5′TTAGCCTTCTTGAGCTTTGAGGTACG3′†  F: GCCGGGGCGAGACCATAGG*  R: CGCACGCGCAAGGCAACC* | 230  253  3352  605 | | 60  60  58  58 |

^†^ (Padmarasu et al. 2018) *- Primers designed for Identification of transformants carrying vector T & C, colony PCR and Rvi12_Cd5 expression analysis*

^§^ (Dalla Costa et al. 2019) *- Primers for determination of CN number of transgene*

^$^(Perini et al. 2014)*- Primers for quantification of endogenous gene expression levels*

*(Herzog et al. 2012)- *Virulence gene for Agrobacterium contamination*
